# Predictors of psychiatric hospitalization during 6 months of maintenance treatment with olanzapine long-acting injection: post hoc analysis of a randomized, double-blind study

**DOI:** 10.1186/1471-244X-13-224

**Published:** 2013-09-16

**Authors:** Haya Ascher-Svanum, Diego Novick, Josep Maria Haro, Jordan Bertsch, David McDonnell, Holland Detke

**Affiliations:** 1 Eli Lilly and Company, Indianapolis, IN, USA; 2 Eli Lilly and Company, Windlesham, Surrey, UK; 3 Departament de Psiquiatria, Universitat Autonoma de Barcelona, Barcelona, Spain; 4 Parc Sanitari Sant Joan de Déu, CIBERSAM, Universitat de Barcelona, Sant Boi de Llobregat, Barcelona, Spain

**Keywords:** Hospitalization, Predictors, Olanzapine long-acting injection, Schizophrenia, Oral olanzapine

## Abstract

**Background:**

Hospitalization is a costly and distressing event associated with relapse during schizophrenia treatment. No information is available on the predictors of psychiatric hospitalization during maintenance treatment with olanzapine long-acting injection (olanzapine-LAI) or how the risk of hospitalization differs between olanzapine-LAI and oral olanzapine. This study aimed to identify the predictors of psychiatric hospitalization during maintenance treatment with olanzapine-LAI and assessed four parameters: hospitalization prevalence, incidence rate, duration, and the time to first hospitalization. Olanzapine-LAI was also compared with a sub-therapeutic dose of olanzapine-LAI and with oral olanzapine.

**Methods:**

This was a post hoc exploratory analysis of data from a randomized, double-blind study comparing the safety and efficacy of olanzapine-LAI (pooled active depot groups: 405 mg/4 weeks, 300 mg/2 weeks, and 150 mg/2 weeks) with oral olanzapine and sub-therapeutic olanzapine-LAI (45 mg/4 weeks) during 6 months’ maintenance treatment of clinically stable schizophrenia outpatients (*n*=1064). The four psychiatric hospitalization parameters were analyzed for each treatment group. Within the olanzapine-LAI group, patients with and without hospitalization were compared on baseline characteristics. Logistic regression and Cox’s proportional hazards models were used to identify the best predictors of hospitalization. Comparisons between the treatment groups employed descriptive statistics, the Kaplan–Meier estimator and Cox’s proportional hazards models.

**Results:**

Psychiatric hospitalization was best predicted by suicide threats in the 12 months before baseline and by prior hospitalization. Compared with sub-therapeutic olanzapine-LAI, olanzapine-LAI was associated with a significantly lower hospitalization rate (5.2% versus 11.1%, *p* < 0.01), a lower mean number of hospitalizations (0.1 versus 0.2, *p* = 0.01), a shorter mean duration of hospitalization (1.5 days versus 2.9 days, *p* < 0.01), and a similar median time to first hospitalization (35 versus 60 days, *p* = 0.48). Olanzapine-LAI did not differ significantly from oral olanzapine on the studied hospitalization parameters.

**Conclusions:**

In clinically stable schizophrenia outpatients receiving olanzapine-LAI maintenance treatment, psychiatric hospitalization was best predicted by a history of suicide threats and prior psychiatric hospitalization. Olanzapine-LAI was associated with a significantly lower incidence of psychiatric hospitalization and shorter duration of hospitalization compared with sub-therapeutic olanzapine-LAI. Olanzapine-LAI did not differ significantly from oral olanzapine on hospitalization parameters.

**Trial registration:**

ClinicalTrials.gov: NCT00088491

## Background

Schizophrenia is a chronic condition in which relapses are likely to occur throughout the patient’s life [1,2]. During a relapse, the patient may need to be admitted to hospital for acute treatment to bring psychotic symptoms under control. Psychiatric hospitalizations and especially re-hospitalizations are linked to poor outcomes and a delayed or reduced likelihood of recovery [3]. In a meta-analysis of published reports on rehospitalization, Weiden and Olfson [4] estimated that 50% of patients treated with typical antipsychotics would be readmitted within 1 year, and about 80% of patients would be readmitted by 2 years. In additional to increasing the personal burden on patients and family members along with disruption of outpatient rehabilitation plans, hospitalization is also costly in economic terms, as the cost of hospitalization accounts for the largest share of treatment costs in schizophrenia [5,6].

Loss (or lack) of medication efficacy and medication non-adherence tend to act synergistically to increase the risk of relapse and hospitalization. Even in patients receiving continuous medication, it has been estimated that 3.5% of patients will relapse per month, and this rate increases to 11% per month among non-adherent patients [3]. Substantial inpatient hospitalization cost savings can thus be realized by linking better pharmacological treatments with more effective strategies to manage medication non-adherence in the management of patients with schizophrenia [3,7]. High severity of positive symptoms, lack of insight, not living with the family, frequent past episodes, addiction to illegal drugs, and global illness severity have all been associated with a higher risk of hospitalization [8]. Suicide behavior, which may be present in over 50% of patients [9] and may cause a 10–13% mortality rate in schizophrenia [10], is also associated with a high risk of hospitalization, as hospitalization is a frequent treatment intervention for suicidal patients.

Prior research has shown differential rates of psychiatric hospitalization among oral antipsychotics, as clozapine and olanzapine were found to be associated with a lower rehospitalization rate compared with other oral antipsychotics [11,12]. Long-acting injection (LAI, depot) antipsychotic formulations are recommended for the treatment of non-adherent patients [13], as depot medications ensure adherence during the injection duration and may help reduce the risk of relapse [14-16]. It has been reported that LAI formulations can reduce the risk of relapse in patients with adherence difficulties [17]. The comparative effectiveness of oral vs. LAI antipsychotics in reducing patients’ relapse and hospitalization rates has been a topic of controversy. A recent literature review and meta-analysis [18] found that LAI antipsychotics were associated with a lower risk of relapse but the limited data on hospitalization did not reveal significant differences between the two formulations. These findings, along with other recent publications [19,20], help highlight that the lack of observed differences in treatment outcomes between oral and LAI antipsychotics may be driven by reliance on randomized clinical trials, which tend not to enroll non-adherent schizophrenia patients, the very group of patients for whom LAI antipsychotics are most appropriate.

The long-acting formulation of olanzapine is available for the treatment of patients with schizophrenia and its safety and efficacy have been shown previously [21,22]. However, no information is available on the predictors of hospitalization during treatment with olanzapine-LAI or how the risk of hospitalization differs between olanzapine-LAI and placebo in the maintenance phase of schizophrenia.

Using data from a 24-week, randomized, double-blind study that compared the safety and efficacy of olanzapine-LAI with oral olanzapine and a placebo-like, sub-therapeutic dose of olanzapine-LAI [22], this post hoc analysis aimed to: (a) identify the predictors of psychiatric hospitalization in the maintenance treatment of schizophrenia patients with olanzapine-LAI, and (b) assess four hospitalization parameters (the prevalence of hospitalization, its incidence rate, duration, and time to first hospitalization) and compare olanzapine-LAI with a placebo-like, sub-therapeutic dose of olanzapine-LAI and oral olanzapine on these hospitalization parameters.

## Methods

### Study design

This multicenter study was conducted by 113 investigators at 112 study sites in 26 countries. The countries which participated in the study were Spain, France, Sweden, Norway, Austria, Belgium, Finland, Netherlands, Germany, Portugal, Italy, Russia, Hungary, Turkey, Greece, Romania, Poland, Israel, Argentina, Brazil, Puerto Rico, United States, Mexico, Australia, South Africa, and Taiwan.

Patients included in the study had a schizophrenia diagnosis according to DSM-IV or DSM-IV-TR and were aged 18 to 75 years. Patients had to have maintained outpatient status and be judged by investigators (based on clinical interview and impression) to have been stable (with respect to their schizophrenia symptoms) for at least 4 weeks before study entry. Patients also had to have Brief Psychiatric Rating Scale (BPRS) positive items ≤4 (on a scale of 1 to 7) at the time of study entry.

The study had an open-label stabilization phase (4–8 weeks) followed by a double-blind randomized phase (24 weeks). During the stabilization phase, all patients were switched to open-label oral olanzapine monotherapy at a dose of 10, 15 or 20 mg/day. Patients who met the stabilization criteria were randomly assigned to double-blind therapy with one of the following five treatments: low-dose olanzapine-LAI (150 mg/2 weeks, *n* = 140), medium-dose olanzapine-LAI (405 mg/4 weeks, *n* = 318), high-dose olanzapine-LAI (300 mg/2 weeks, *n* = 141), oral olanzapine (a stabilized dose of oral olanzapine, 10, 15 or 20 mg/day *n* = 322) and a sub-therapeutic (very low) dose of olanzapine-LAI (45 mg/4 weeks, *n* = 144).

Patients were assessed using the Positive and Negative Syndrome Scale (PANSS) [23], the Clinical Global Impressions-Severity of Illness scale (CGI-S) [24], the Health Outcomes Form [25] (which assesses work status, living conditions, functional activities and suicide threats), the Drug Attitude Inventory (DAI-10) [26], the Hospitalization Inventory, and the Medical Outcomes Study Short-Form 36-item version (SF-36) [27].

The study protocol was approved by institutional review boards at each site. After receiving a complete description of the study, all patients and/or their authorized legal representatives provided written informed consent before participation. A complete description of the methods can be found in the main study publication [22].

### Variables

Patients who were included in the double-blind, randomized phase and who had baseline data and at least one post-baseline assessment during follow up were included in the analysis (*n* = 1064). Data for patients in the three olanzapine-LAI active medication groups (low-dose olanzapine-LAI, medium-dose olanzapine-LAI, and high-dose olanzapine-LAI) were pooled into a single olanzapine-LAI group. The duration of each psychiatric hospitalization (based on data in the Hospitalization Inventory) was calculated as the discharge date minus the admission date plus one.

Patients with and without a psychiatric hospitalization during the study period were first compared on the following 16 baseline variables: sex, age, age at illness onset, any suicide threat before baseline (in the 12 months before baseline), PANSS total score, CGI-S score, DAI total score, working for pay, living independently, functional impairment, geographical region, tobacco use, body mass index, psychiatric outpatient visits before baseline, health related quality of life (SF-36 score), and the presence of a prior hospitalization.

### Statistical analysis

The *p* values for group comparisons were calculated using the Chi-square test for categorical variables and the Wilcoxon-Mann and Whitney test for numerical variables.

The Kaplan–Meier product limit estimation method was used to compare time to first hospitalization for the three treatment groups. Regression models were built to identify the baseline factors associated with subsequent psychiatric hospitalization; a logistic regression model was used for analysis of psychiatric hospitalization during the study period, and Cox’s proportional hazards model was used for analysis of time to first psychiatric hospitalization.

The regression models included as covariates the baseline variables that were found to differ significantly between patients with psychiatric hospitalization versus those without psychiatric hospitalization in the descriptive analysis. These variables were: sex, age of the patient, any suicide threat before baseline, previous hospitalization, age at illness onset, PANSS total score, and DAI total score. To take into consideration variations in healthcare utilization across regions, the regression models were adjusted by geographical region (Western Europe, Eastern Europe, America, Other).

All analyses were performed using SAS, version 9.1 (SAS Institute, Inc., Cary, NC).

## Results

Patient characteristics at baseline by treatment group are summarized in Table 1. Of the 1064 patients included in this study, 598 were treated with olanzapine-LAI, 322 with oral olanzapine and 144 with sub-therapeutic olanzapine-LAI. There were no statistically significant differences between the groups on any of the baseline sociodemographic or clinical characteristics.

**Table 1:** **Baseline characteristics by treatment group**^**a**^

**Characteristic**	**Olanzapine-LAI (*n *= 598)**	**Sub-therapeutic olanzapine-LAI (*n *= 144)**	**Oral olanzapine (*n *= 322)**
**Male (%)**	65.2	66.7	64.9
**Working for pay (%)**	20.5	18.1	14.1
**Living independently (%)**	21.2	17.4	18.8
**At least one functional activity (%)**	83.6	77.1	83.8
**Any suicide threat (%)**	3.4	5.6	1.9
**Prior psychiatric hospitalization (%)**	4.68	6.94	4.97
**Current tobacco use (%)**	48.1	49.3	49.8
**Geographical region (%)**			
America	29.8	31.3	31.4
Eastern Europe	30.1	29.2	29.2
Western Europe	29.8	27.8	30.1
Other	10.4	11.8	9.3
**Age in years, mean (SD)**	38.8 (11.1)	39.5 (11.6)	39.0 (11.6)
**Age at onset, years, mean (SD)**	25.6 (8.0)	26.1 (9.2)	25.6 (8.4)
**Body mass index (BMI), kg/m**^**2**^**, mean (SD)**	26.5 (5.1)	26.8 (5.2)	26.4 (5.1)
**PANSS total, mean (SD)**	55.4 (15.5)	57.8 (15.9)	56.1 (15.6)
**CGI-S score, mean (SD)**	3.1 (0.9)	3.2 (0.9)	3.1 (1.0)
**DAI score, mean (SD)**	7.4 (1.8)	7.3 (1.9)	7.5 (1.7)
**SF-36 physical score, mean (SD)**	49.4 (8.2)	48.5 (8.7)	49.7 (8.1)
**SF-36 mental score, mean (SD)**	42.7 (11.1)	42.9 (11.8)	43.1 (11.9)
**Psychiatric outpatient visits before baseline, mean (SD)**	10.2 (10.1)	9.5 (10.2)	9.7 (10.4)

*CGI-S* Clinical Global Impression-Severity, *DAI* Drug Attitude Inventory, *LAI* long-acting injection, *PANSS* Positive and Negative Symptom Score, *SF-36* Medical Outcomes Study Short-Form 36-item version, *SD*, standard deviation.

^a^All *p* values for treatment group comparisons were greater than 0.05.

Among all study participants, the percentage of hospitalized patients was slightly higher for males than females (5.8% versus 5.5%, not significant). The mean age of the patients who were hospitalized was 42.1 (SD 9.6) years compared with 38.8 (SD 11.4) years for those not hospitalized (*p* = 0.02). Mean age of onset was very similar at 25.6 years (SD 7.4) for those hospitalized versus 25.7 years (SD 8.4) for those not hospitalized. The mean PANSS total score of the hospitalized group was 52.2 (SD 16.2) compared with 56.1 (SD 15.5) for the non-hospitalized group (*p* = 0.05). Mean CGI-S scores for patients with versus without hospitalization were nearly identical; 3.08 (SD 0.9) for patients who were hospitalized and 3.07 (SD 0.9) for those who were not, while mean DAI scores of the hospitalized group were 7.9 (SD 1.6) versus 7.4 (SD 1.8) for those without hospitalization (*p* = 0.01). In terms of type of olanzapine therapy, the rate of hospitalization was higher for patients in the sub-therapeutic olanzapine-LAI (11.1% hospitalized) compared with olanzapine-LAI and oral olanzapine groups (5.2% and 4.0%, respectively) (*p* < 0.01). A total of 14.7% of patients who had threatened suicide were hospitalized compared with 5.3% of those who did not threaten suicide (*p* = 0.04). Finally, previous hospitalization was strongly related to hospitalization during follow up; the percentage of patients admitted during follow up was 4.6% among patients with no previous hospitalizations and 24% among patients with previous hospitalizations (*p* < 0.001).

**Figure 1:**
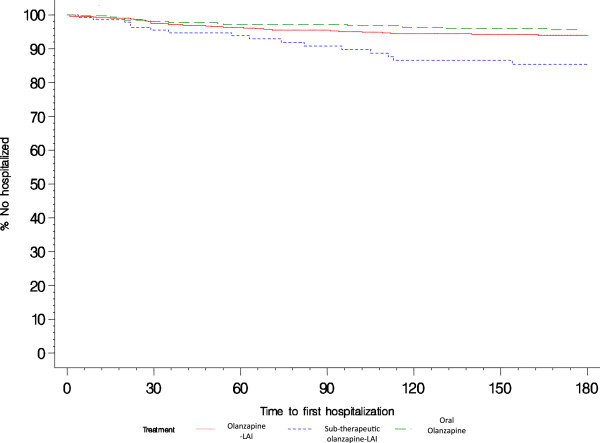
Time (days) to psychiatric hospitalization by treatment group: olanzapine-LAI, sub-therapeutic olanzapine-LAI, and oral olanzapine.

Table 2 presents the hospitalization parameters by treatment group. Compared with sub-therapeutic olanzapine-LAI, olanzapine-LAI was associated with a significantly lower hospitalization rate (5.2% versus 11.1%, *p* < 0.01), a lower mean number of hospitalizations (0.1 versus 0.2, *p* = 0.01), and a shorter mean duration of hospitalization (1.5 days versus 2.9 days, *p* < 0.01). The treatment groups did not differ significantly on median time to first hospitalization among hospitalized patients (35 days for sub-therapeutic olanzapine-LAI versus 60 days for olanzapine-LAI, *p* = 0.48). Olanzapine-LAI did not differ significantly from oral olanzapine on the studied hospitalization parameters. The Kaplan-Meier figure (Figure 1) shows there was little difference between olanzapine-LAI and oral olanzapine in the percentage of patients hospitalized and time to first hospitalization. In contrast, the percentage of patients hospitalized was higher and time to first hospitalization was shorter among patients treated with sub-therapeutic olanzapine-LAI.

**Table 2:** Psychiatric hospitalization parameters by treatment group

**Hospitalization parameter**	**Olazapine-LAI**	**Sub-therapeutic olanzapine-LAI**	**Oral olanzapine**	***p *value**^**a**^		
				**Olanzapine-LAI versus sub-therapeutic olanzapine-LAI**	**Olanzapine-LAI versus oral olanzapine**	**Oral olanzapine versus sub-therapeutic olanzapine-LAI**
**Hospitalization rate, %**	5.2	11.1	4.0	0.0088	0.4369	0.0035
**Number of hospitalizations, mean (SD)**	0.1 (0.4)	0.2 (0.6)	0.1 (0.5)	0.0092	0.4385	0.0038
**Number of days hospitalized, mean (SD)**^**b**^	1.5 (12.26)	2.9 (15.03)	2.3 (17.2)	0.0084	0.4639	0.0041

*CGI-S* Clinical Global Impression-Severity, *DAI* Drug Attitude Inventory, *PANSS* Positive and Negative Symptom Score, *LAI* long-acting injection, *SD* standard deviation.

^a^*p* values calculated using Chi-square and t-tests.

^b^Calculation of the mean number of days includes both patients hospitalized and not hospitalized (calculation is number of days in the hospital including all patients divided by total number of patients in the group).

The findings of the regression models are presented in Table 3. Baseline factors that were significantly associated with hospitalization in the olanzapine-LAI group were: prior suicide threats with an odds ratio (OR) of 7.1 (95% CI: 2.2; 23.0), and previous hospitalization with an OR of 5.84 (95% CI: 1.92; 17.78).

**Table 3:** Findings from regression models: baseline variables associated with psychiatric hospitalization

**Factors associated with hospitalization during follow up**	**Treatment group/regression model**		
	**Olanzapine-LAI/logistic regression**	**All patients/logistic regression**	**All patients/Cox regression**
**Oral olanzapine**	NA	0.78 (0.39; 1.58)	0.75 (0.39; 1.45)
**Sub-therapeutic olanzapine-LAI**	NA	2.34 (1.18; 4.65)	2.41 (1.29; 4.52)
**Female**	0.81 (0.35; 1.87)	0.77 (0.42; 1.42)	0.70 (0.39; 1.23)
**Age**	0.99 (0.94; 1.05)	1.01 (0.98; 1.05)	1.02 (0.98 ;1.05)
**Suicide threats**	7.09 (2.19; 23.01)	3.63 (1.26; 10.46)	3.76 (1.44; 9.83)
**Previous hospitalization**	5.84 (1.92; 17.78)	5.54 (2.50; 12.27)	5.18 (2.62; 10.21)
**Age at onset**	1.03 (0.97; 1.09)	1.02 (0.99; 1.06)	1.02 (0.99; 1.05)
**Total PANSS score**	0.98 (0.96; 1.01)	0.98 (0.96; 0.99)	0.98 (0.96; 0.99)
**DAI score**	0.91 (0.72; 1.15)	1.12 (0.93; 1.35)	1.10 (0.93; 1.32)

Values are odds ratio (95% confidence interval).

*DAI* Drug Attitude Inventory, *PANSS* Positive and Negative Symptom Score, *LAI* long-acting injection, *NA* not applicable.

The Cox regression model found that patients receiving sub-therapeutic olanzapine-LAI had a significantly shorter time to first psychiatric hospitalization compared with patients receiving olanzapine-LAI (hazard rate 0.75 versus 2.41). Other statistically significant baseline factors associated with a shorter time to hospitalization among patients in the olanzapine-LAI group were higher PANSS score, prior suicide threats and previous psychiatric hospitalization.

## Discussion

This post hoc analysis found that during the 6-month maintenance treatment of schizophrenia patients with olanzapine-LAI, psychiatric hospitalization was best predicted by suicide threats at baseline and by prior psychiatric hospitalization. Suicide threats were strongly associated with future hospitalization even after taking patients’ history of previous hospitalization into account. Current findings are consistent with prior research showing that suicide threats are one of the main causes of hospitalization in schizophrenia patients [28] and tend to predict future hospitalization [29].

As expected, past hospitalization was strongly associated with future hospitalization. A number of other schizophrenia studies have also found this association [30-32], reflecting a known general phenomenon where past behavior tends to be a robust predictor of future behavior [33].

In the current analysis, symptom severity at baseline was only weakly associated with the risk of hospitalization. Although some studies have found symptom severity at baseline to be associated with hospitalization, others have not [1]. It is possible that findings differ because the association depends on the point at which symptoms are assessed in the course of the illness; in the current study, patients were assessed when their symptoms were relatively mild and clinically stable.

As for the study’s second objective, this post hoc analysis found that compared with placebo-like sub-therapeutic olanzapine-LAI, treatment with olanzapine-LAI was associated with a significantly lower rate of psychiatric hospitalization (5.2% versus 11.1%), a lower incidence of hospitalization during follow up (0.1 versus 0.2) and a shorter duration of hospitalization among hospitalized patients (1.5 days versus 2.9 days). This finding is consistent with a recent meta-analysis that found that treatment with antipsychotics confers a significantly greater relapse prevention compared with placebo in the treatment of schizophrenia [34].

The risk of hospitalization – as assessed here using four hospitalization parameters – however, was not found to differ significantly between the oral and LAI formulations of olanzapine. This finding is likely driven by the study design, a randomized clinical trial (RCT) of stable, mildly ill and mostly adherent outpatients. In this sample, the hospitalization rate was relatively low, which lowers the power of finding significant findings. Although non-adherent patients are typically the ones chosen for depot formulations in clinical practice, they are often reluctant to enroll in RCTs. This interpretation seems consistent with a recent study that found differences in effectiveness when comparing results from RCTs and observational studies, with observational studies finding larger medication differences [20]. A large review of RCTs comparing oral with LAI treatment found significant differences in relapse rates between RCTs of depot and oral antipsychotics, but did not find differences in hospitalization rates or other outcome parameters [18]. As mentioned above, naturalistic studies have shown differences in hospitalization rates between oral and depot formulations. For example, Tiihonen and colleagues conducted retrospective analyses of Finnish databases and found depot therapy to be associated with a significantly lower risk of hospital admission compared with oral formulations of the same compounds [11,12]. In another similar study in Hungary, depot antipsychotics were found to have a lower hospitalization rate than many other oral antipsychotics [19]. Similar findings were observed in the Schizophrenia Outpatients Health Outcomes (SOHO) Study [35], a large pan-European, naturalistic, prospective, observational study of schizophrenia patients. That analysis of SOHO focused on non-adherent patients who were initiated on typical antipsychotics in oral or depot formulations (*n* = 431) and found that patients initiated on depot formulations had a significantly lower rate of hospitalization and a lower mean number of hospitalizations following 6 months of treatment. The potential confounding impact of clinical trials versus naturalistic practice settings will require further research to clarify the relative advantage of depot versus oral atypical antipsychotics in reducing the risk of hospitalization among patients with schizophrenia. Observational studies may be better suited to study the impact of depot therapy on treatment outcomes among non-adherent patients because these patients tend not to participate in RCTs.

Current findings need to be evaluated in the context of the study limitations. Firstly, this post hoc analysis used data from an RCT with the primary objective of comparing olanzapine-LAI, oral olanzapine and sub-therapeutic olanzapine-LAI on time to exacerbation of symptoms of schizophrenia in clinically stable patients who were first stabilized on oral olanzapine [22]. Thus, as all patients were first stabilized on oral olanzapine and their baseline PANSS scores were within the mildly ill range, the chances of finding differences between the oral and depot formulations of olanzapine were much reduced. This may also explain the relatively low hospitalization rate observed in the study. Moreover, participants in the depot treatment arm were randomly assigned to switch from oral olanzapine to olanzapine-LAI, whereas those in the oral treatment arm got to stay on their preferred stabilized doze of oral olanzapine, suggesting that study design may have been biased against depot patients. Secondly, this study (like most RCTs) included a low percentage of patients who were non-adherent with treatment, although these are typically the target patients for depot therapy in usual care. Thus these findings may not generalize to treatment with olanzapine-LAI in naturalistic care settings.

## Conclusion

In this 6-month maintenance study, psychiatric hospitalization was best predicted by previous suicide threats and prior psychiatric hospitalization, two predictors that were previously observed with oral antipsychotic therapy in the treatment of schizophrenia. In addition, olanzapine-LAI therapy was found to be associated with a significantly lower psychiatric hospitalization rate, a lower mean number of hospitalizations, and a shorter mean hospitalized duration compared with sub-therapeutic olanzapine-LAI. Olanzapine-LAI did not differ significantly from oral olanzapine on these hospitalization parameters, likely reflecting the impact of the study’s design.

## Competing interests

This study was funded by Eli Lilly and Company. JMH acted as a consultant or speaker for Astra-Zeneca, Eli Lilly, Lundbeck, and Roche. JB conducted the statistical analysis under a contract between Fundació Sant Joan de Déu and Eli Lilly and Company. HA-S, DN, DMcD and HD are employees of Eli Lilly and Company.

## Authors’ contributions

HA-S conceived of the study, participated in its design, the analytical plan, the interpretation of the results, and helped write the manuscript. DN and JMH participated in the design of the study, the analytical plan, the interpretation of the results, and assisted in drafting the manuscript. JB participated in the design of the study and the analytical plan, and performed the statistical analyses. DMcD and HD participated in the interpretation of the results and assisted in drafting the manuscript. All authors read and approved the final manuscript.

## Pre-publication history

The pre-publication history for this paper can be accessed here:

http://www.biomedcentral.com/1471-244X/13/224/prepub
